# “But man is not made for defeat”: insights into Ernest Hemingway’s dementia

**DOI:** 10.1590/0004-282X-ANP-2021-0299

**Published:** 2022-01-31

**Authors:** Léo Coutinho, Hélio Afonso Ghizoni Teive

**Affiliations:** 1 Universidade Federal do Paraná, Serviço de Neurologia, Unidade de Distúrbios de Movimento, Curitiba PR, Brazil. Universidade Federal do Paraná Serviço de Neurologia Unidade de Distúrbios de Movimento Curitiba PR Brazil; 2 Universidade Federal do Paraná, Programa de Pós-Graduação em Medicina Interna e Ciências da Saúde, Grupo de Doenças Neurológicas, Curitiba PR, Brazil. Universidade Federal do Paraná Programa de Pós-Graduação em Medicina Interna e Ciências da Saúde Grupo de Doenças Neurológicas Curitiba PR Brazil

**Keywords:** History of Medicine, Neurology, Art, Dementia, Neurodegenerative Diseases, História da Medicina, Neurologia, Arte, Demência, Doenças Neurodegenerativas

## Abstract

Ernest Hemingway is widely regarded as one of the greatest fiction writers of all time. During his life, he demonstrated several signs of psychological suffering with gradual worsening and presentation of cognitive issues over his late years. Some of his symptoms and the course of his disease suggest that he might have suffered from an organic neurodegenerative condition that contributed to his decline, which culminated in his suicide in 1961. In this historical note, we discuss diagnostic hypotheses compatible with Hemingway’s illness, in light of biographical reports.

## RESUMO

Ernest Hemingway é considerado um dos escritores mais lidos de todos os tempos. Durante sua vida, ele demonstrou diversos sinais de sofrimento psicológico com piora gradual durante seus últimos anos, associado à apresentação de distúrbios cognitivos. Alguns de seus sintomas, assim como o curso da doença, sugerem que ele talvez tenha padecido de uma condição neurodegenerativa orgânica que contribuiu para o seu declínio, culminando em seu suicídio em 1961. Nesta nota histórica, discutimos hipóteses diagnósticas compatíveis com a doença de Hemingway, à luz de relatos biográficos.

## INTRODUCTION

**Figure 1.**
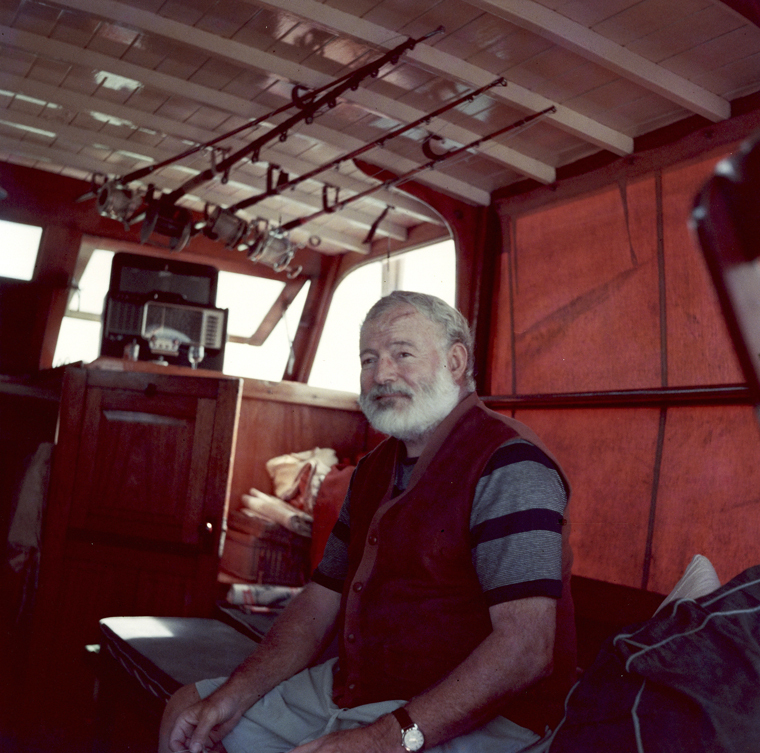
Ernest Hemingway in the cabin of his boat,*Pilar* (circa 1950s).

Source: Ernest Hemingway Collection. John F. Kennedy Presidential Library and Museum, Boston. Licensed under a Public Domain Mark.

Ernest Hemingway (1899-1961) (Figure 1) is one of the world's most praised literary writers. His objective prose created masterpieces such as *For whom the bell tolls* and *The old man and the sea*^1^^-^^4^. 

Born in 1899 in Oak Park, Illinois, Hemingway began writing in journalism and war correspondence. He married four times and had three children. 

Hemingway was given the ultimate accolades in literature: the Pulitzer in 1953 and the Nobel Prize in 1954^5^^,^^6^. Despite an achieved life, Hemingway presented signs of psychiatric suffering^7^^-^^12^, which culminated in his suicide in 1961 in Ketchum, Idaho. Recent evidence suggests that in his late years he presented neurological signs attributable to dementia^3^.

At the 60^th^ anniversary of Hemingway’s death, we discuss his neurological condition, emphasizing organic hypotheses based on his biographic reports. 

## COGNITIVE AND BEHAVIORAL DECLINE

**Figure 2.**
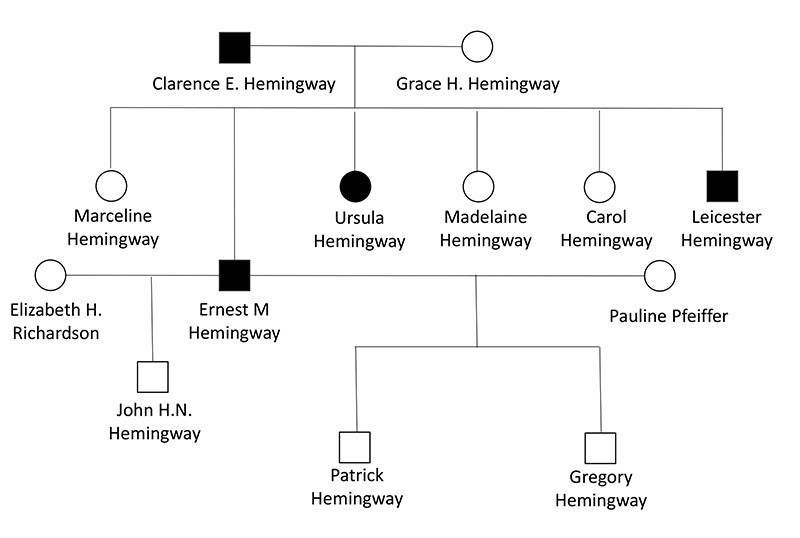
Pedigree chart for the Hemingway family, showing first-degree relatives to Ernest^3^^,^^7^. Icons in black represent family members who committed suicide. Marriages with Martha Gellhorn and Mary Welsh were omitted, as they did not have children.

Hemingway presented early signs of a psychiatric condition, possibly bipolar disorder, with documented maniac and depressive episodes, in addition to a significant family history of suicide (Figure 2), although only his father had committed suicide before him^1^^-^^3^^,^^7^. Many patients with this condition share outstanding creativity^7^^,^^8^. Patients with bipolar disorder have an increased dementia risk with an incidence of 25.2% in a recent cohort^9^. Previous papers detailed his psychological ailment^10^^-^^13^.

The following evidence supports the presence of dementia:

(1) Hemingway’s decline was inexorable, despite electroconvulsive therapy carried in the Mayo Clinic.
(2) In his last years, his cognition sharply declined, impairing his writing.
(3) He presented risk factors for dementia, namely alcoholism, sexual risk behavior, and repeated head trauma^3^.

The precise onset of Hemingway’s decline is unclear, but it possibly initiated during his fifth decade of life^1^^-^^3^. The disease was marked by a primacy of behavioral symptoms with late cognitive issues, raising several hypotheses (Table 1)^1^^-^^3^.

**Table 1.** Diagnostic possibilities compatible with Ernest Hemingway’s condition and epidemiology.

Condition	Arguments in favor	Arguments against
Behavioral variant Frontotemporal Dementia (bvFTD)	Disinhibition. Early onset. Late cognitive disability.	Absence of other features (lack of empathy, obsessive behavior, problems in executive function).
Lewy Body Dementia (LBD)	Psychosis. Delusions. Fluctuations.	Absence of Parkinsonism. Lack of well systematized hallucinations.
Vascular dementia	Multiple risk factors. Family history of vascular. complications.	No history of strokes.
Chronic traumatic encephalopathy (CTE)	Multiple concussions. He was a notorious brawler. Predominantly behavioral presentation.	None.
Alcohol toxicity/Vitamin deficiency	Heavy alcohol consumption. Other documented complications of alcohol intake (hepatopathy, withdrawal syndrome).	Lack of early amnestic symptomatology.
Neurosyphilis	Risk sexual behavior.	Lack of other neurosyphilis hallmarks (motor symptoms, cranial nerve palsy). Lack of a well-documented primary treponemal infection.
Huntington’s disease (HD)	Phenotypes with pure behavioral/cognitive symptomatology.	Rare presentation. No members of the Hemingway family presented motor phenomenology compatible with HD.

Hemingway experienced progressive disinhibition. He would often say inappropriate things during social gatherings and engage in more sexually liberated experiences. This disinhibition can be perceived in the sexual themes present in his last works, *A moveable feast* and G*arden of Eden*^1^^-^^3^. The former cruelly depicts his first two wives and his friendship with Scott Fitzgerald, possibly motivated by disinhibition.

His paranoia and delusions increased, with a belief that he was under FBI surveillance. Other sources of paranoia were his hypochondria, fear of impoverishment, and the possibility of arrest for illegal hunting and for “taking liberties with a minor”^1^^-^^3^.

Hemingway would present frequent and unpredictable bursts of aggressiveness, particularly towards his last wife, Mary, who endured significant abuse^1^^-^^3^.

After his first admittance to the Mayo Clinic (1960), Hemingway presented amnestic symptomatology. This would be a burden to his writing, and he would consider finishing *A moveable feast* impossible. He needed help with the manuscript revision from his wife and editor and his last works would be published only posthumouslly^1^^-^^3^.

## DISCUSSING THE POSSIBILITIES

### Frontotemporal dementia (FTD)

Hemingway´s clinical features are compatible with the behavioral variant of frontotemporal dementia (bvFTD). FTD presents an earlier onset than other neurodegenerative etiologies, as seen in the writer’s case. Genetics plays a significant role in FTD, and up to 40% of patients present a family history of dementia. However, other bvFTD features, such as lack of empathy, obsessive behavior, and a dysexecutive syndrome were not present^14^.

### Lewy body dementia (LBD)

Hallucinations and psychosis with marked fluctuations are the hallmarks of LBD^15^. This diagnosis was recently proposed as etiology for Hemingway’s decline^3^. However, parkinsonism was never described in his case. Moreover, Hemingway’s psychosis presented more delusions than well-substantiated hallucinations, making LBD an unlikely diagnosis^1^^-^^5^. 

### Vascular dementia

In the Mayo Clinic, Hemingway was diagnosed with severe hypertension, prediabetes, and dyslipidemia. He was under suspicion of hemochromatosis, but a liver biopsy was contraindicated considering his precarious health^1^^-^^5^. Hemingway had a family history of vasculopathy, particularly related to diabetes^1^^-^^5^. 

Although Hemingway’s biography reports no strokes, these comorbidities are risk factors for small vessel disease and subcortical ischemic vascular dementia. As clinical presentation is variable and overlapping with other dementia etiologies is common, this is a consistent hypothesis^16^. 

### Chronic traumatic encephalopathy (CTE)

Hemingway endured nine major head traumas during his war service, including a mortar blast. In 1954, he survived two plane crashes. He practiced football and boxing from an early age, acted as an amateur bullfighter, and was a reckless driver^1^^-^^5^. CTE is a plausible hypothesis, as this condition often presents with behavioral symptomatology, particularly aggressiveness and mood changes, while cognition is of late affection^17^.

### Alcohol toxicity/Vitamin deficiency

To say Hemingway was a heavy drinker would be an understatement. He spent a significant part of his time in Havana at the bar *La Floridita*, being served with *Papa Dobles* (the Hemingway daiquiri) by the bartender^2^^,^^3^. The role of alcohol consumption in dementia is documented, being a risk factor for vascular dementia and Alzheimer’s disease. The proposed mechanisms include direct neuronal toxicity and secondary vitamin deficiencies^18^.

### Neurosyphilis

Hemingway had multiple sexual partners, including extramarital relationships. Although he was at risk for syphilis, a more diverse clinical picture would be expected. The absence of motor symptomatology, cranial nerve palsy, and other hallmarks of neurosyphilis, besides the absence of a well-documented primary treponemal infection, make this diagnosis improbable^19^.

### Huntington’s disease (HD)

Certain phenotypes of HD have a predominance or exclusivity of non-motor symptomatology, presenting behavioral and cognitive symptoms, such as the exhibited in Hemingway’s case and family history; motor phenomena may have a late onset or never occur. However, this is a rare presentation and an unlikely hypothesis. Phenotypic variability occurs within the same family, and other members of the Hemingway family would present motor symptomatology^20^.

In conclusion, Hemingway’s case would remain a challenge in modern days. His personality traits would pose an obstacle for the detection of behavioral symptoms of neurodegeneration.

Although a psychiatric condition is acknowledged, Hemingway’s symptomatology is compatible with organic dementia. In the author’s opinions and in accordance with recent literature^3^, bvFTD and CTE, possibly associated with a vascular component, might have contributed to his decline. 

Remarkably, Hemingway tried to write to his very end despite his cognitive impairment; a display of tenacity worthy of Santiago, the main protagonist of *The old man and the sea*.
